# QSAR based model for discriminating EGFR inhibitors and non-inhibitors using Random forest

**DOI:** 10.1186/s13062-015-0046-9

**Published:** 2015-03-25

**Authors:** Harinder Singh, Sandeep Singh, Deepak Singla, Subhash M Agarwal, Gajendra P S Raghava

**Affiliations:** Bioinformatics Center, Institute of Microbial Technology, Sector 39-A, Chandigarh, India; Institute of Cytology and Preventive Oncology, Sector 39, Noida, 201301 Uttar Pradesh India

**Keywords:** EGFR inhibitors, Classification of EGFR inhibitors and non-inhibitors, Active substructure, Active functional groups, PubChem fingerprint, QSAR, Random forest

## Abstract

**Background:**

Epidermal Growth Factor Receptor (EGFR) is a well-characterized cancer drug target. In the past, several QSAR models have been developed for predicting inhibition activity of molecules against EGFR. These models are useful to a limited set of molecules for a particular class like quinazoline-derivatives. In this study, an attempt has been made to develop prediction models on a large set of molecules (~3500 molecules) that include diverse scaffolds like quinazoline, pyrimidine, quinoline and indole.

**Results:**

We train, test and validate our classification models on a dataset called EGFR10 that contains 508 inhibitors (having inhibition activity IC_50_ less than 10 nM) and 2997 non-inhibitors. Our Random forest based model achieved maximum MCC 0.49 with accuracy 83.7% on a validation set using 881 PubChem fingerprints. In this study, frequency-based feature selection technique has been used to identify best fingerprints. It was observed that PubChem fingerprints FP380 (C(~O) (~O)), FP579 (O = C-C-C-C), FP388 (C(:C) (:N) (:N)) and FP 816 (ClC1CC(Br)CCC1) are more frequent in the inhibitors in comparison to non-inhibitors. In addition, we created different datasets namely EGFR100 containing inhibitors having IC_50_ < 100 nM and EGFR1000 containing inhibitors having IC_50_ < 1000 nM. We trained, test and validate our models on datasets EGFR100 and EGFR1000 datasets and achieved and maximum MCC 0.58 and 0.71 respectively. In addition, models were developed for predicting quinazoline and pyrimidine based EGFR inhibitors.

**Conclusions:**

In summary, models have been developed on a large set of molecules of various classes for discriminating EGFR inhibitors and non-inhibitors. These highly accurate prediction models can be used to design and discover novel EGFR inhibitors. In order to provide service to the scientific community, a web server/standalone EGFRpred also has been developed (http://crdd.osdd.net/oscadd/egfrpred/).

**Reviewers:**

This article was reviewed by Dr Murphy, Prof Wang and Dr. Eisenhaber.

**Electronic supplementary material:**

The online version of this article (doi:10.1186/s13062-015-0046-9) contains supplementary material, which is available to authorized users.

## Background

Epidermal Growth Factor Receptor (EGFR) is a member of the receptor tyrosine kinase family. It is involved in the regulation of several critical processes such as cell proliferation, survival, adhesion, migration and differentiation [1,2]. It is one of the most studied cancer drug target [3], whose aberrant activity has been associated with a number of cancers [4]. Since, inhibition of EGFR has been demonstrated to have therapeutic potential. Thus, a large number of tyrosine kinase inhibitors have been designed in past [5]. The treatment of patients with EGFR based inhibitors as targeted therapy thus has shown a significant reduction in the cancer progression. As a result, a large number of researchers have continuously synthesized small molecules and investigated them for anti-EGFR activity using a variety of *in vitro* cellular and enzymatic assay systems. This has resulted in the identification of a range of bioactive compounds making a large volume of biological and structural information available in the public domain. These hundreds of small molecules belong to various distinct chemical classes such as pyrimidine, quinazoline and indole. Although, the number of active EGFR inhibitors is steadily expanding, yet the search for newer EGFR inhibitors is still a significant scientific challenge.

In the recent years, various structure and ligand-based approaches like virtual screening [6], molecular docking [7], QSAR [8,9] and pharmacophore modeling [10] have been widely exploited for identifying new EGFR inhibitor molecules. QSAR models generated in the past have been developed using single scaffold based analogues along with experimental data generated by a single bioassay system [11-14]. These models have been developed on a limited set of molecules for a particular class, and thus the predictive coverage is limited. Thus, there is a need to develop a single model that can cover wide ranging inhibiting molecules from various classes of chemicals. Unique model for diverse molecules is also important in identification of chemical component/properties (e.g., structural-fragments) that contribute to inhibitory bioactivities of EGFR inhibitors. In the present study, we have used a large dataset of ~3500 diverse molecules for understanding structure-activity relationship and for developing QSAR-based prediction models. We develop models using various machine-learning techniques (e.g., random forest) for predicting inhibition potential of a molecule. We identify important scaffolds/substructures/fingerprints that play a significant role in discrimination in EGFR inhibitors and non-inhibitors. As the coverage of chemical space offered by this model is large, for this reason the application of this system is expected to be high.

## Results

### Frequency of functional groups

**Figure 1:**
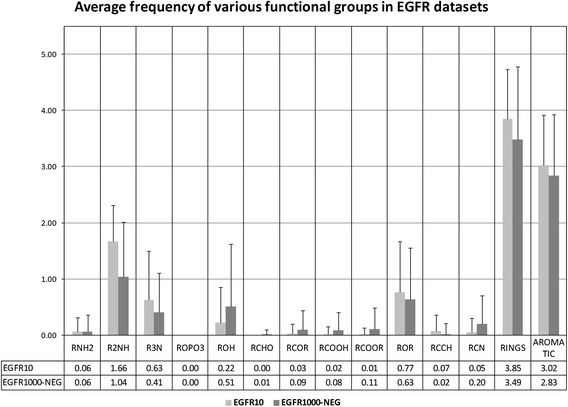
**Average frequency with standard deviation of various functional groups in inhibitors and non-inhibitors of EGFR10 and EGFR1000 datasets respectively.**

**Figure 2:**
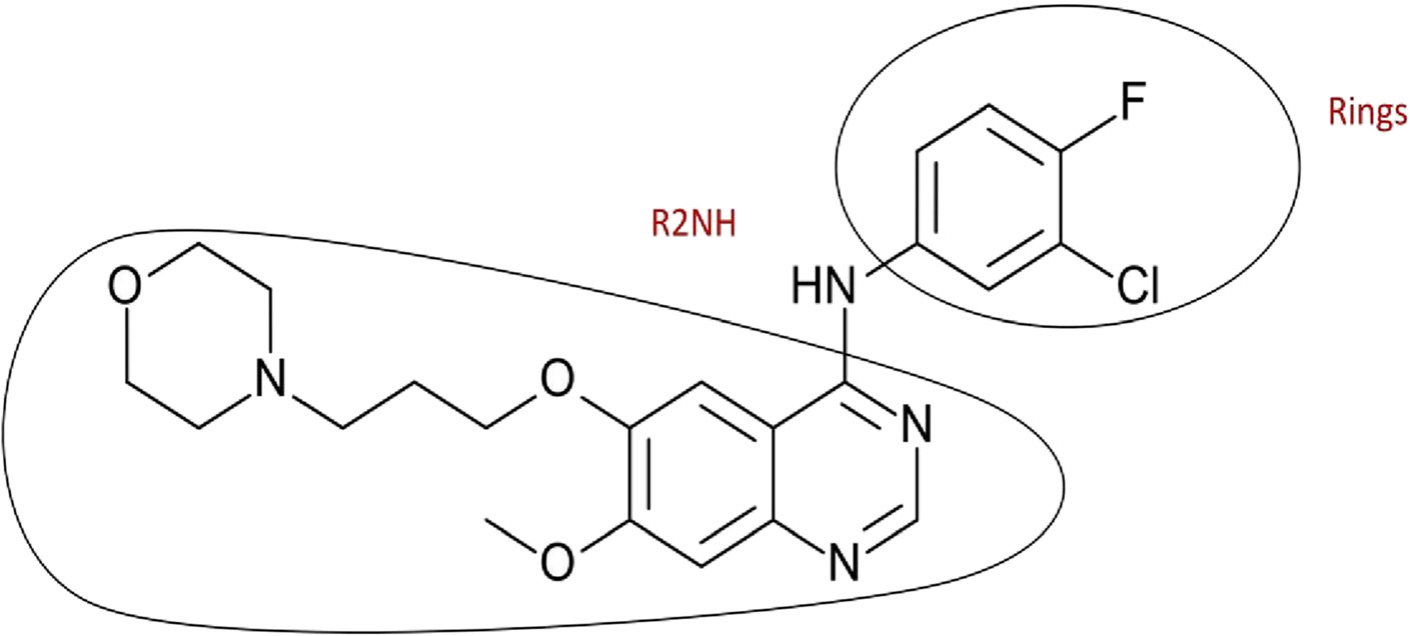
**Shows EGFR inhibitor gefitinib marked with two frequently occurring functional groups (R2NH and rings).**

We used chemmineR [15] to calculate the various functional groups frequency in EGFR10 inhibitors and EGFR1000 non-inhibitors (inhibitors having IC_50_values greater than 1000 nM). We observe from the functional group frequency distribution that the number of the secondary amines (R2NH), tertiary amines (R3N), and rings are higher in the most active EGFR inhibitors (Figure 1). Almost all the 4-anilino quinazoline based EGFR small molecule kinase inhibitors that compete for ATP binding site contains this functional group (R2NH). On one side of Nitrogen is the core group, which is responsible for making hydrogen bonds with EGFR active site residues while on the other side, stabilizing group is present that extends into the cleft for tighter interactions with the enzyme. It is in accordance with the known biological information that the most active EGFR inhibitors like gefitinib drug demonstrate the above characteristics (Figure 2). Thus, it indicates that use of the above functional groups, as backbone moiety is helpful for designing inhibitors active against EGFR.

### Maximum common substructures (MCS)

**Figure 3:**
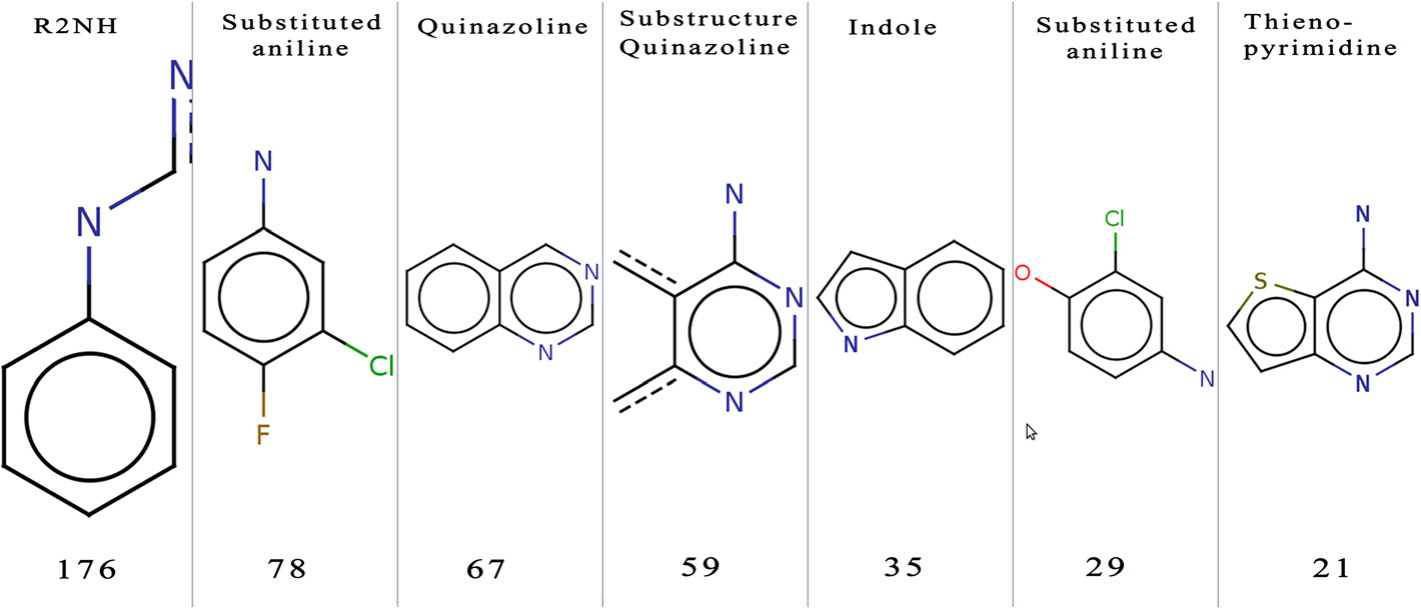
**Maximum common substructures (MCS) and their count found in active/inhibitors of EGFR10 dataset.**

The MCS module of Chemaxon (http://www.chemaxon.com/) was used to find the maximum common substructures in EGFR10 inhibitor dataset. We mainly find that three structural scaffolds (4-anilino quinazoline, indole and anilino thienopyrimidine) dominate within the dataset (Figure 3). The presence of 4-anilino quinazoline substructures is as per the expectation, as these are present in known drugs gefitinib and erlotinib. Consequently, chemists worldwide have been synthesizing, and testing analogues having these moieties to identify new molecules with higher potency. In addition, in the previous studies, analogues of anilino thienopyrimidines have been generated and demonstrated activity in the low nanomolar range against EGFR [16-18]. We also find substituted anilines (halogenated anilines) that are present in EGFR inhibitors like gefitinib, EKB-569, lapatinib and act as linkers have high frequency of occurrence (Figure 3). As depicted in Figure 3, we observe that the 1st substructure is aniline that is attached to the quinazoline in Anilino-quinazoline, while the 2nd substructure occurs in two known EGFR inhibitors gefitinib, EKB-569. The 3rd substructure is quinazoline ring; 5th substructure is indole, and the 6th substructure is a part of Lapatinib, a well known EGFR inhibitors. This analysis gives us indication that new analogues synthesized using these skeletons would have a better probability of exhibiting significant binding interactions and activity against EGFR.

### Analysis of fingerprints

**Figure 4:**
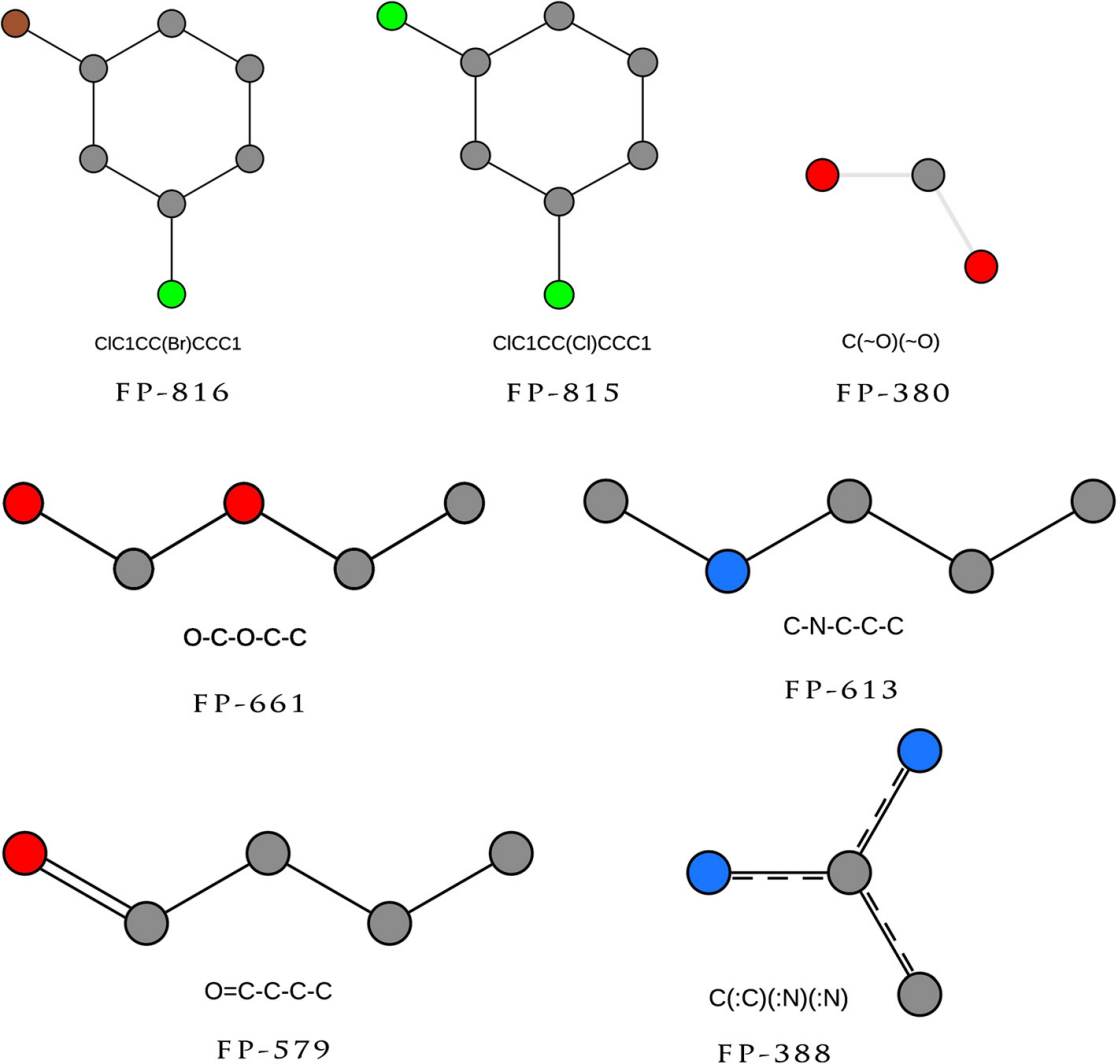
**Structural representation of PubChem fingerprints found more frequently in inhibitors as compare to non-inhibitors.**

Using our fingerprint selection approach we found that PubChem fingerprint (FP) 816 (ClC1CC(Br)CCC1), FP815 (ClC1CC(Cl)CCC1), FP380 (C(~O) (~O)), FP579 (O = C-C-C-C), FP388 (C(:C) (:N) (:N)), FP661 (C-C = C-C-C) and FP613 (C-N-C-C-C) are among the best fingerprints for discrimination of EGFR inhibitors as opposed to non-inhibitors (Table 1). As evident from Figure 4, PubChem FP816 and PubChem FP815 are highly similar cyclic structure with bromine or chlorine substitution at ortho and para position indicating that the presence of halogens attached to a cyclic structure influences the activity against EGFR. The PubChem FP186 state the presence of > = 2 saturated or aromatic carbon-only ESSSR (canonic Extended Smallest Set of Smallest Rings) ring size 6. We also observed high frequency of PubChem FP388 substructure of 4- anilinoquinazoline in active inhibitors. A number of derivatives of 4- anilinoquinazoline are inhibitors of EGFR [19-21]. The PubChem FP661, FP613 and FP579 are in essence part of the ring structure, which appeared to be aliphatic in nature. It is interesting to note that best positive fingerprints are non-aromatic and aliphatic in nature.

**Table 1:** **List of best 10 positive and negative fingerprints that occurs more frequently in inhibitors and non-inhibitors of EGFR10 dataset respectively.**

**Best 10 positive fingerprints**				**Best 10 negative fingerprints**			
**Fingerprint Number**	**Freq. (+)**	**Freq. (−)**	**Diffe-rence**	**Fingerprint Number**	**Freq. (+)**	**Freq. (−)**	**Diffe-rence**
380	71.85	43.64	28.21	698	21.26	45.15	−23.89
579	75.79	52.82	22.97	673	8.27	31.80	−23.53
189	38.78	17.35	21.43	690	57.48	76.51	−19.03
388	67.52	46.41	21.11	700	19.29	38.00	−18.71
816	24.21	6.24	17.97	714	3.54	20.42	−16.88
815	32.68	16.68	15.99	145	30.31	45.28	−14.96
374	39.96	27.06	12.90	701	14.37	28.50	−14.13
613	32.87	20.95	11.92	669	2.17	15.92	−13.75
661	31.50	19.82	11.68	195	6.50	18.02	−11.52
348	40.16	29.50	10.66	382	2.56	11.61	−9.05

### Classification based on best fingerprints

**Figure 5:**
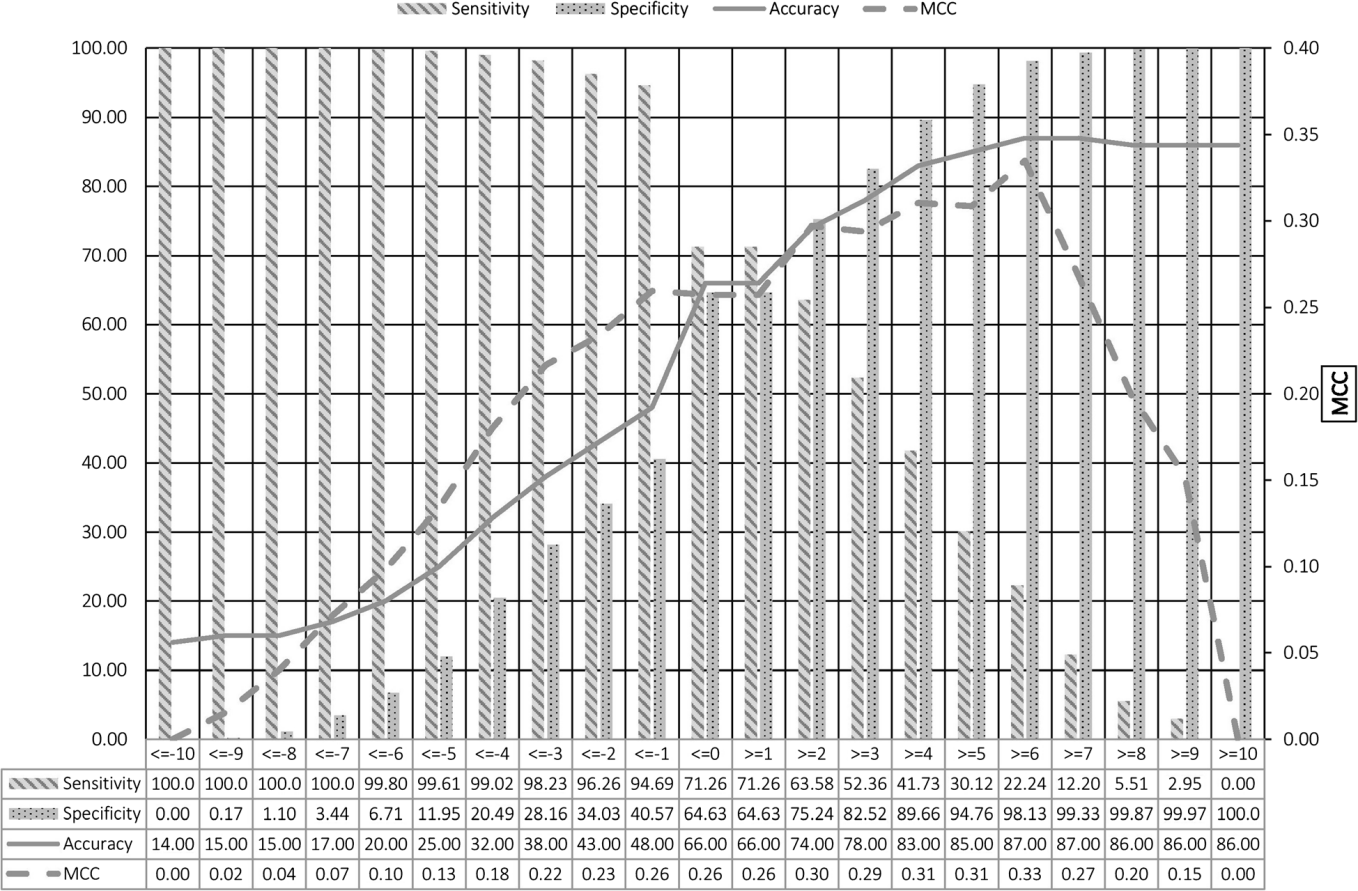
**The performance of simple method that predicts inhibitors based on occurrence of best 20 fingerprints found in inhibitors and non-inhibitors.** The secondary Y-axis shows the range of MCC and X-axis shows the summed up values of best 20 Fingerprints.

In order to understand, whether combining the best fingerprints for classification purpose would increase the efficiency of the classification or not. Therefore, we have combined the best 10 positive and best 10 negative to make best 20 fingerprints as described in Additional file 1: Table S2, S3. For each compound the best 20 fingerprints were summed, accordingly a compound having zero score has equal number of positive and negative fingerprints. If a compound has > = 1 score, positive fingerprints dominate over negative fingerprints, and we classify the compound as EGFR inhibitor. Similarly, for the compound having score below zero means negative fingerprints dominate over positive fingerprints and the compound was classified EGFR non-inhibitor. It was observed that neither positive nor negative fingerprints achieved a reasonable sensitivity and specificity. The balance results were obtained at score < =0 and > =1, where equal number of positive and negative fingerprints were present. We obtained 71.7% sensitivity, 64.6% specificity, 66.0% accuracy and 0.26 MCC (Figure 5). Thus, it can be concluded that by combining best positive and best negative fingerprints, reasonable sensitivity and specificity can be achieved.

### Model developed on EGFR10 dataset

We developed classification models for predicting inhibitors using various algorithms/techniques that include IBK, Bayes, Naive Bayes, SVM, and Random forest. These models were evaluated using fivefold cross-validation and 881 PubChem fingerprints. It was observed that model based on Random forest algorithm using 100 trees performed best among various classifiers and achieved accuracy 84.95% with MCC 0.49 (Table 2). Random forest based models were developed using 881 fingerprints and evaluated on the EGFR10 validation set and achieved accuracy 83.65% with MCC 0.49 MCC (Table 3). The performance of models developed using best 10–100 fingerprints is slightly lower than model developed using 881 fingerprints. The algorithm of Random forest learn the best fingerprints itself and the model developed using 881 fingerprints perform better.

**Table 2:** **The performance of models based on various classifiers developed & evaluated on EGFR10 dataset**

**Classifier**	**Sensitivity**	**Specificity**	**Accuracy**	**MCC**	**ROC**
IBK	68.69	84.98	82.63	0.45	0.87
Bayes	68.73	70.57	70.31	0.29	0.72
Naive Bayes	69.87	67.96	68.23	0.27	0.70
SVM	67.11	86.24	83.48	0.46	0.87
Random Forest	68.74	87.67	84.95	0.49	0.89

**Table 3:** **The performance of models developed on EGFR10 dataset, class-specific molecules and EGFR10 excluding single class, evaluated using cross-validation techniques for testing on same-class of molecules**

**Trained on**	**Tested on**	**Sensitivity**	**Specificity**	**Accuracy**	**MCC**	**ROC**
EGFR10 train	EGFR10 train	68.74	87.67	84.95	0.49	0.89
EGFR10 train	EGFR10 Validation	69.89	86.03	83.66	0.49	0.89
Pyrimidine	Pyrimidine	69.25	92.13	86.92	0.62	0.92
Pyrimidine	Quinazoline	68.62	54.88	58.88	0.21	0.67
Quinazoline	Quinazoline	68.15	79.63	76.31	0.45	0.81
Quinazoline	Pyrimidine	67.86	64.04	64.91	0.27	0.74
EFGR10-Pyrimidine	EFGR10-Pyrimidine	68.7	94.08	91.34	0.59	0.92
EFGR10-Quinazoline	EFGR10- Quinazoline	69.66	96.4	94.04	0.64	0.95
EFGR10- Pyrimidine	Pyrimidine	68.06	76.74	74.77	0.4	0.77
EFGR10- Quinazoline	Quinazoline	60.31	76.25	71.66	0.35	0.72

### Class specific models

In addition, we made an attempt to develop models for predicting class-specific inhibitors. We developed models on Pyrimidine for predicting Pyrimidine based inhibitors. These models were evaluated on Pyrimidine class of molecules using five-fold cross validation technique and achieved maximum accuracy 86.92 with MCC 0.62 (Table 3). These models also evaluated on Quinazoline class of molecules and achieved poor accuracy 58.88 with MCC 0.21. Similarly we developed models on Quinazoline dataset and achieved maximum accuracy 76.31 with MCC 0.45, when evaluated on Quinazoline class of molecules. These models perform poor with MCC 0.27, when evaluated on Pyrimidine class of molecules. It is clear from results shown in Table 3, that models developed on class-specific molecules are only suitable for that class of molecules but not suitable for other class of molecules.

In order to understand the performance of general classifier for prediction of unknown EGFR class, we developed two datasets: EGFR10-pyrimidine (all molecules except Pyrimidine class of molecules) and EGFR10-quinazoline (all molecules except Quinazoline class of molecules). The model trained and tested on EGFR10-pyrimidine and EGFR10-quinazoline dataset achieved accuracy 91.34 with MCC 0.59 and accuracy 94.04 with MCC 0.64 respectively (Table 3). Next, we evaluate our general classifier (trained on EGFR10-pyrimidine dataset) on Pyrimidine class of molecules and achieved accuracy 74.77 with MCC 0.40. Similarly, we also evaluated our general classifier (trained on EGFR10-quinazoline dataset) on quinazoline dataset and achieved accuracy 71.66 with MCC 0.35 MCC (Table 3).

### Models developed on additional datasets

In addition to EGFR10 dataset, we also developed and evaluated our models on additional datasets EGFR100 and EGFR1000. Initially, models developed and evaluated on EGFR100 train set using 881 fingerprints achieved maximum MCC 0.58 (Table 4). We also evaluate prediction performance on EGFR100 validation set and achieved MCC 0.58 using 881 fingerprints (Table 4). Similarly, models were developed and evaluated on EGFR1000 train and validation sets (Table 4). We achieved MCC more than 0.6 for both train and validation sets. It is important to note that performance was better for EGFR1000 dataset in comparison to other datasets; it is probably due to balancing of number of inhibitors and non-inhibitors data.

**Table 4:** **The performance of models developed on EGFR100 and EGFR1000 train sets using different PubChem fingerprints and evaluated on validations sets**

**Trained on**	**Tested on**	**Fingerprints count**	**Sensitivity**	**Specificity**	**Accuracy**	**MCC**	**ROC**
EGFR100 train set	EGFR100 train set	PubChem 881	88.01	73.34	78.2	0.58	0.90
EGFR100 train set	EGFR100 validation set	PubChem 881	91.1	68.5	76.8	0.58	0.90
EGFR1000 train set	EGFR1000 train set	PubChem 881	86.97	78.36	82.92	0.66	0.89
EGFR1000 train set	EGFR1000 validation set	PubChem 881	85.7	85.5	85.6	0.71	0.90

#### Comparison with existing methods

It is necessary to compare performance of newly developed method with existing methods in order to justify whether newly developed method is worth. Unfortunately, it is not possible for us to compare our method with existing methods as we developed models on the largest dataset. In addition, our models are classification models whereas models developed in previous studies are regression-based models. None of the previous methods used more than 200 molecules for developing models whereas we used around 3500 molecules.

#### Web server and standalone

In this study, we developed a user-friendly web server for prediction anti-EGFR molecules. The user can either draw a single compound or provide a list of compounds in SMILES format for virtual screening. The web server allows users to generate analogs based upon a combination of given scaffold, building blocks and linkers. The server subsequently predicts the potential EFGR inhibitors of analogs. The output shows the classification of the query compound as anti-EGFR inhibitor or non anti-EGFR inhibitor along with the probability score depending upon the model selected. For the prediction of large number of molecules, we have also provided Python and R language based standalone package.

## Conclusion

Epidermal growth factor protein is a well-known cell surface receptor protein involved in cancer. Numerous models have been developed in the past that considers few molecules of a similar nature identified using a single bioassay system. As these models have limitations, it has become necessary to develop a model that considers heterogeneous dataset of molecules covering broad chemical space so that the pace of EGFR inhibitor drug discovery is accelerated. In this study, we have used a large dataset of diverse molecules from published literature to develop an integrated robust and accurate prediction model using 881 PubChem fingerprints. In addition, analysis of fingerprints displays the contribution of a particular pattern towards anti-EGFR activity. Our analysis suggests that PubChem FP 816 (ClC1CC(Br)CCC1), FP815 (ClC1CC(Cl)CCC1), FP380 (C(~O) (~O)), FP579 (O = C-C-C-C), FP388 (C(:C) (:N) (:N)), FP661 (C-C = C-C-C) and FP613 (C-N-C-C-C) are important for anti-EGFR activity. In this paper, we have introduced a novel frequency based approach for selection of most relevant binary fingerprints. Additionally, a freely available web server and standalone package named EGFRpred (http://crdd.osdd.net/oscadd/egfrpred) has been designed for prediction of anti-EGFR inhibitors. Overall, this study will be helpful in the area of computational designing of novel anti-EGFR molecules used for cancer treatment.

## Methodology

### Dataset

We obtained 3528 anti-EGFR compounds along with their inhibitory concentration (*IC*_*50*_) from database EGFRindb that covers around 350 research articles [22]. These compounds are diverse in nature and belong to various structural scaffolds. Based on the inhibition activity, three different datasets were constructed, i.e. EGFR1000, EGFR100 and EGFR10 datasets (Additional file 1: Table S1). In the case of EGFR10 dataset, a compound is assigned as inhibitor or active molecule if IC_50_ (50% inhibition) is less than 10 *nM*. EGFR10 dataset contains 508 inhibitors and 2997 non-inhibitors (IC_50_ > 10 *nM*). In addition, we created EGFR100 dataset where a compound is classified as inhibitor if IC_50_ is less than 100 *nM* otherwise non-inhibitor. Similarly, we created EGFR1000 dataset where a compound is classified as inhibitor if IC_50_ is less than 1000 *nM* otherwise non-inhibitor. In this study, we obtained inhibition activity (IC_50_ of molecules) from various studies/assays, and it was observed that only a few molecules have multiple IC_50_ values. We removed all those compounds having conflicting inhibition IC_50_ values, for example, in case of EGFR10 we removed 23 compounds having IC_50_ values less than 10 *nM* as well as greater than 10 *nM*. In the case of EGFR100 and EGFR100 datasets, we removed 16 compounds and 22 compounds respectively. For evaluating the performance of the model, we created two types of set from above datasets called train and validation set. For example EGFR10 dataset is split into two set called EGFR10 train set consist of 90% of data and a set consist of remaining 10% of data called EGFR10 validation set.

All molecules in EGFR10 dataset were examined and observed two classes of molecules (Pyrimidine and Quinazoline) dominate that dataset. Thus, we created two datasets from EGFR10 dataset called Pyrimidine and Quinazoline. The Pyrimidine dataset consists of 246 Pyrimidine inhibitors and 838 Pyrimidine non-inhibitors. In case of Quinazoline, there are 218 Quinazoline inhibitors and 540 Quinazoline non-inhibitors. In order to understand the performance of general classifier on unknown class, we created two additional datasets EGFR10-pyrimidine dataset and EGFR10-quinazoline dataset. The EGFR10-pyrimidine dataset consist of 262 EGFR inhibitors and 2162 non-inhibitors. There are no pyrimidine inhibitors in the complete EGFR10-pyrimidine dataset. Similarly, we also created EGFR10-quinazoline dataset consist of 290 EGFR inhibitors and 3000 non-inhibitors.

### Descriptor calculation

Chemical descriptors are the representative features of chemical molecules that are responsible for its activity. In this study, we have used PubChem based 881 binary fingerprints calculated using PaDEL software [23]. The complete details of PubChem 881 fingerprints along with a description are available from PubChem website (ftp://ftp.ncbi.nlm.nih.gov/pubchem/specifications/pubchem_fingerprints.txt). Here, we describe the use of a frequency-based approach for selection of highly significant descriptors.

### Descriptor selection using FREQ_a-i_ based approach

We used a simple frequency-based approach for selection of best fingerprints. For each descriptor or fingerprint, the frequency of a descriptor, in active and inactive molecules, is calculated using Equation 1 and 2.

1 $$ {\mathrm{F}}_{\mathrm{i}}^{\mathrm{A}}=\frac{{\displaystyle {\sum}_{\mathrm{j}=1}^{\mathrm{NA}}}{\mathrm{D}}_{\mathrm{i}}^{\mathrm{j}}}{\mathrm{NA}}\times 100 $$

2 $$ {\mathrm{F}}_{\mathrm{i}}^{\mathrm{A}}=\frac{{\displaystyle {\sum}_{\mathrm{j}=1}^{\mathrm{NA}}}{\mathrm{D}}_{\mathrm{i}}^{\mathrm{j}}}{\mathrm{NA}}\times 100 $$

Where ${\mathrm{F}}_{\mathrm{i}}^{\mathrm{A}}$ and ${\mathrm{F}}_{\mathrm{i}}^{\mathrm{A}}$ represent mean of i^th^ fingerprint in active (A) and inactive (I) molecules respectively. NA and NI are the total number of molecules in active and inactive datasets respectively. ${\mathrm{D}}_{\mathrm{i}}^{\mathrm{j}}$ is the value of i^th^ fingerprint for j^th^ molecule (value is either 0 or 1). Finally, we compute fingerprint score (FS) of each fingerprint using following Equation 3.

3 $$ {\mathrm{F}\mathrm{S}}_{\mathrm{i}}={\mathrm{F}}_{\mathrm{i}}^{\mathrm{A}}-{\mathrm{F}}_{\mathrm{i}}^{\mathrm{I}} $$

Where FS_i_ is the inhibitory score of i^th^ fingerprint. The descriptors having higher positive score FS means there are more preferred in active molecules as comparison to inactive molecules. Similarly, a higher negative score states that the fingerprint is more preferred in inactive molecules (or not preferred in active molecules). Magnitude of a fingerprint score represents significance of fingerprint.

In order to select best fingerprints, first we remove all redundant/similar fingerprints having correlation greater than 0.6, using software package RapidMiner [24] and obtained 465 non-redundant fingerprints. Secondly, we select 20 best descriptors from 465 fingerprints, 10 having highest positive score (highly preferred in active molecules) and 10 having highest negative score (highly preferred in inactive molecules).

### Classification

In this study, we have used various classifiers implemented in WEKA package and SVM^*light*^ [25,26]. Further, based on results and computational efficiency we selected Random forest classifier for the final prediction. The final models were developed using Random forest algorithm implemented in WEKA package [27,28]. A Random forest is a classifier consisting of a collection of tree-structured classifiers {*h*(**x**, Θ_*k*_), *k* = 1,…} where the {Θ_*k*_} are independent identically distributed random vectors and each tree casts a unit vote for the most popular class at input **x**” [27].

### Performance evaluation

The performance of the models was evaluated using five-fold cross-validation techniques. In this technique, training and testing were carried five times in such a way that each time one set was used for testing and remaining (*n-1)* sets were used for training. The train set was further randomly divided into five training and testing sets. To avoid any bias in the prediction model, an independent validation set was also used for further evaluation. The whole process was repeated five times, and the results were reported after obtaining the average. Finally, fitness of the model was assessed using various standard parameters like sensitivity, specificity, accuracy, and Matthew’s correlation coefficient (MCC) [29].

## Reviewer’s comment

### Response to Dr Murphy

**Question 1:** The authors describe using standard compound features and machine learning techniques to train models of the relationship between chemical structure and EGF receptor inhibition activity. This best way to treat this task is as a regression problem, in which the task is to predict the activity of a given compound, not whether it is ?active? or ?inactive? using an arbitrary threshold.

**Response:** We agree with the reviewer that regression based models provides more information than classification based models. In previous studies, also regression models have been developed for predicting EGFR inhibitors (PLoS ONE 2014. 9(7): e101079). In order to develop a regression models, one must collect data for a single class of molecules whose activity has been determined using the same bioassay system. Due to these limitations, existing methods have been developed on limited set of molecules (maximum 200 molecules). In this study, our aim is to develop robust models on a large set of EGFR inhibitors. Here, we developed prediction models on ~3,500 EGFR inhibitors obtained from different studies. On this large dataset, where molecules belong to different classes and has IC_50_ from varied bioassay system, thus it is not feasible to develop a regression method. This is the reason we developed classification models in this study instead of regression models.

**Question 2:** No attempt is made to determine whether the classifier generalizes well across different structures (e.g., functional groups). An even stronger approach would be to hold out an entire functional group during training and determine whether the resulting classifier can generalize to the held out group.

**Response:** We are grateful to the reviewer for above suggestion. Now, we have developed class/function specific models and evaluate their performance on self-class of molecules and other class of molecules. It is clear from results that class-specific molecules are suitable only for that class of molecules whereas our model works equally well for all class of molecules. In our revised version of manuscript, we have described limitations and strength of function or class specific models.

**Question 3:** The manuscript refers to ?validation? datasets but does not describe their composition or whether the compounds in the validation sets were included in the feature selection step. This is only made clear in Figure S1.

**Response:** In revised version of manuscript, we have clearly described training and validation sets.

**Question 4:** It is unclear why feature selection was performed before training the random forest classifiers, since the random forest should learn the most useful feature combinations. The results in fact show that the best performance is achieved using all of the features. Thus the manuscript should be simplified by removing feature selection.

**Response:** The main reason for feature selection is to select best fingerprints. The best fingerprints performance is comparable to the performance achieved using all fingerprints. The importance of selecting best fingerprint is to help biologist/chemist in understanding and designing inhibitors, considering the best fingerprint structure. It is always advisable to develop models on minimum number of descriptors in order to avoid over fitting.

**Question 5:** No information is provided about the parameters used for the random forest classifier training.

**Response:** In this study, we used the Random Forest implemented in WEKA package and found that using 100 trees we achieved the best performance. In revised manuscript, parameter used for developing models has been revealed.

**Question 6:** There are a number of English errors in the manuscript. For example, ?biasness? is not considered to be a valid English word by most scholars. ?Bias? is the correct term. ?Accessed? is used but ?assessed? is meant.

**Response:** In the revised manuscript, we have incorporated reviewer suggestion and improved the overall language of the manuscript.

Comments from second revision

**Question 1:** My second question was not addressed. The question is not whether one can train structural class-specific classifiers; that has been done before. The question is whether if one trains a general classifier but holds out all members of a specific structural class while doing so, the resulting classifier does well at predicting activities for the held-out class. They have not answered this question.

**Response:** We apologize that we were not able to understand reviewer’s previous query completely. In this version of manuscript, we have tried to address above query. In order to address above query we create two datasets EGFR10-pyrimidine (all molecules except pyrimidine class of molecules) and EGFR10-quinazoline (all molecules except Quinazoline class of molecules). It means EGFR10-pyrimidine dataset consist of all EGFR inhibitors and EGFR non-inhibitors, except Pyrimidine class of molecules. We developed model using EGFR10-pyrimidine dataset and tested/validated this model on Pyrimidine class of molecules. We achieved accuracy of 74.77 with MCC 0.40 on Pyrimidine class of molecules. Similarly, we also developed model on EGFR10-quinazoline dataset and tested on Quinazoline class of molecules; we achieved accuracy 71.66 with MCC 0.35 MCC (Table 3). These results have been included in revised version.

**Question 2:** The authors apparently missed the point in my fourth question about feature selection. The data that feature selection is unnecessary since not using it gives better results. In that case it does not seem warranted to do feature selection at all.

**Response:** We agree with the reviewer that model developed using selected features is not giving better performance. Thus, in the revised manuscript, we have removed the section describing the performance of models developed using best features.

**Question 3:** Regarding my fifth question, the answer is now provided about how many trees were trained. However, this raises a more serious problem of potential overestimation of generalizability if the validation set was used in part or in whole for any decision-making. For example, if the number of trees was chosen to give the best performance on the validation set, any reported accuracies using that number of trees are likely overestimates of the generalizability (the accuracy expected on future data).

**Response:** We are thankful to reviewer for raising important query on overestimation of models performance. In this study, the optimized number of trees were obtained from dataset used for training; estimated during development of models using five-fold cross validation. Best models on training dataset were evaluated on validation dataset. In simple words, we have not used validation or independent dataset for training or estimation of optimized number of trees.

Response to Prof Wang

Strong points:

1. This manuscript describes the training and evaluation of a classifier of EGFR inhibitors vs non-inhibitors. This is a useful application.
2. It can make classification of a broad range of molecules. This generality is good.

### Weak points

**Question 1:** The individual steps and the overall methodology are rather standard. So once the training/testing data is collected, it is just mechanically feeding into a standard classifier learning and testing process. So the methodological novelty is limited.

**Response:** The novelty of the work is that for the first time we have tried to develop a generalized QSAR model for classifying EGFR inhibitors from non-inhibitors. We identified fingerprints frequently occurs in inhibitors and non-inhibitors in order to identify best descriptors for developing classification models. We tried wide range of algorithms and techniques for developing models for searching best techniques. In our revised version, we also described class specific models developed for predicting inhibitors of specific class.

**Question 2:** The performance does not look exciting to me (~70% sensitivity, ~86% specificity). No effort is made to seriously improve on it. Also, the performance analysis lacks depth; e.g., it is unclear which classes of molecules are predicted more accurately and why.

**Response:** We agree with reviewer that performance is not very impressive; it is because it includes different class of molecules. After getting comment of reviewer, we perform more analysis and develop class-specific models. In the revised manuscript, we have updated the analysis section and added one section on class specific models.

**Question 3:** There is no quantitative comparison with existing works. So superiority of the proposed classifier is unproven. I understand that these works may be specific to a class of molecules. You can still compare with them by restricting your test set to those classes of molecules. Your classifier can be trained on the full training set (or on a class-specific subset of the training set) and tested on a specific class. The competitive classifier can be trained on class-specific subset of the training set and tested on the specific class. 

**Response:** We agree that no comparison with the existing methods was mentioned in the manuscript. We had searched the literature and found no classification method for distinguishing EGFR inhibitors from non-inhibitors exist. The existing methods developed in recent years are based upon small dataset and are regression models. As suggested by the reviewer, we have developed the class specific models and found that Pyrimidine class of molecules is most accurately predicted. In revised manuscript, we have added one section on comparison with existing methods.

**Question 4:** Judging from the best features, they appear in both inhibitors and non-inhibitors, though there is some difference in frequency. The difference in frequency is not exploited. Each feature can be weighted based on the difference in its frequency in inhibitor and non-inhibitor. Some alternative classifier models that can take advantage of such weights can be considered (e.g., bagging of naïve Bayes based classifiers).

**Response:** We used frequency of features in inhibitors and non-inhibitors and exploited it for selecting best features. These features have been used for developed highly accurate classification models; revised manuscript includes detail description. In this study, we selected positive fingerprints that are more frequent in inhibitors as compare to non-inhibitors. Similarly, we selected negative fingerprints that are more frequent in non-inhibitors as compare to inhibitors. Based upon the frequency difference we selected best60 fingerprint and developed a model, that achieved 0.49 MCC and the model developed using all 881 fingerprints also achieved 0.49 MCC. We also developed models using bagging of naïve Bayes based classifiers in WEKA package. The performance of the naïve Bayes classifier improved from 0.27 MCC to 0.30 MCC, still the performance is poor as compare to Random forest.

**Question 5:** Currently, molecules from different classes are mixed. It may be worth considering each class (pyrimidine, quinazoline, etc.) separately, and have several class-specific classifiers, in addition to an undifferentiated classifier. Given a new molecular, if a class-specific classifier for its class is available, the class-specific classifier is used. Otherwise, the undifferentiated classifier is used.

**Response:** We are grateful to the reviewer for the suggestion. We have developed class specific models and the results have been reported in the section “Class specific Models”. The models for two largest classes Pyrimidine and Quinazoline have been developed as suggested by reviewer and implemented in the web server. The user can select either class specific models or the generalized model.

**Question 6:** While some fingerprints are highlighted, other than their presence in known EGFR inhibitors, they are not analyzed/discussed informatively within the context of EGFR. One should discuss their structural/physical/chemical significance, after all the structures of EGFR and some of these inhibitors/non-inhibitors are known.

**Response:** In our revised version, we have briefly discussed the functional group, maximum common substructure and analysis of fingerprints. In the updated manuscript, we added more information in context of EGFR (Figure 3).

**Question 7:** The supporting data needs to be prepared in a way that is more convenient for others to repeat the study or make comparative study.

**Response:** The supporting data is updated and available on the website in smiles format.

Comment from second revision

Deficiencies:

1. The value of this work is mostly in the collection of training/testing data. The methodological novelty is very limited in the developed classifiers.
2. The performance of the developed classifiers is not impressive. Given the lack of methodological novelty, I feel that more effort needs to be devoted to improve classifier performance and utility. I understand it is hard to achieve improvement through methodological novelty. However, you can still improve the utility of the classifiers by more mechanical means. Class-specific classifier is one possible ---I am glad to see that tried. One can also create a family of classifiers with different trade-off between sensitivity and specificity, and let user choose high-sensitivity or high-specificity classifier to use.

**Response:** We are grateful for appreciating our efforts and for providing useful suggestions. Based on reviewer’s suggestion, we incorporated threshold parameter with models in our web server. This threshold selection will allow users to predict inhibitors with high specificity by selecting higher threshold and better coverage/sensitivity by selecting lower threshold.

**Question 1:** It seems that you have the selection of the top (50) fingerprints before hand. In selecting these fingerprints, have you used the test data? In a cross-validation setting, fingerprints must be selected fresh from the training portion of that cross-validation fold. Otherwise, the obtained accuracy/sensitivity/etc. is not an acceptable estimate of the performance of the resulting classifier. This makes your validation methodology unsound

**Response:** The best fingerprints were selected only from the training set; test/validation set was not used for selection of best fingerprints.

### **Response to Dr. Eisenhaber**

**Question:** Report form: I think the authors provide a useful software application tailored to the design of EGFR inhibitors. It is especially laudable that the authors provide both download for datasets and software. It would be of interest to which extent the tools are applicable if mutated forms of EGFR are to inhibited.

**Answer:** It’s difficult to say how accurately EGFRpred models can predict inhibitors for mutant EGFR. Recently our group developed a web server ntEGFR (http://crdd.osdd.net/oscadd/ntegfr) for predicting inhibitors against wild and mutant EGFR that allows users to predict inhibitors that inhibit mutant form of EGFR. In this study, we have developed generalized method for predicting inhibitors against EGFR not specifically against mutant form of EGFR.

## Additional file

Additional file 1: Table S1. Distribution of data in different datasets. **Table S2.** Best 100 positive Fingerprints in EGFR10, 100 and 1000 datasets. **Table S3.** Best 100 negative Fingerprints in EGFR10, 100 and 1000 datasets.
