# Supplementary material for: QSAR based model for discriminating EGFR inhibitors and non-inhibitors using Random forest
Source: Biol Direct. 2015 Mar 25;10:10. doi: 10.1186/s13062-015-0046-9 (PMC4372225; doi:10.1186/s13062-015-0046-9)
Supplement: Additional file 1: Table S1. — Distribution of data in different datasets. Table S2. Best 100 positive Fingerprints in EGFR10, 100 and 1000 datasets. Table S3. Best 100 negative Fingerprints in EGFR10, 100 and 1000 datasets. [file 13062_2015_46_MOESM1_ESM.docx]

Table S1: Distribution of data in different datasets

| **Dataset** | **Total** | **Positive** | **Negative** |
| --- | --- | --- | --- |
| **EGFR1000** | 3506 | 1854 | 1652 |
| **EGFR100** | 3512 | 1166 | 2346 |
| **EGFR10** | 3510 | 510 | 3001 |

TableS2:**Best 100 positive Fingerprints in EGFR10, 100 and 1000 datasets**

| **EGFR10 Dataset** | | | | **EGFR100 Dataset** | | | | **EGFR1000 Dataset** | | | |
| --- | --- | --- | --- | --- | --- | --- | --- | --- | --- | --- | --- |
| ***FP No.*** | ***Freq. (+)*** | ***Freq. (-)*** | ***Difference*** | ***FP No.*** | ***Freq. (+)*** | ***Freq. (-)*** | ***Difference*** | ***FP No.*** | ***Freq. (+)*** | ***Freq. (-)*** | ***Difference*** |
| 380 | 71.85 | 43.64 | 28.21 | 623 | 73.02 | 44.92 | 28.11 | 623 | 69.42 | 37.37 | 32.05 |
| 579 | 75.79 | 52.82 | 22.97 | 815 | 35.57 | 11.02 | 24.55 | 815 | 29.72 | 7.27 | 22.45 |
| 189 | 38.78 | 17.35 | 21.43 | 348 | 44.24 | 24.77 | 19.48 | 786 | 29.77 | 11.27 | 18.51 |
| 388 | 67.52 | 46.41 | 21.11 | 579 | 69.24 | 49.87 | 19.37 | 348 | 39.81 | 21.68 | 18.12 |
| 816 | 24.21 | 6.24 | 17.97 | 786 | 31.87 | 15.67 | 16.20 | 579 | 64.72 | 46.76 | 17.97 |
| 815 | 32.68 | 16.68 | 15.99 | 289 | 31.01 | 16.78 | 14.23 | 807 | 27.13 | 10.30 | 16.83 |
| 374 | 39.96 | 27.06 | 12.90 | 190 | 29.38 | 16.05 | 13.33 | 190 | 28.21 | 13.63 | 14.58 |
| 613 | 32.87 | 20.95 | 11.92 | 654 | 31.01 | 17.81 | 13.21 | 730 | 21.95 | 7.63 | 14.32 |
| 661 | 31.50 | 19.82 | 11.68 | 568 | 30.41 | 17.93 | 12.48 | 760 | 43.80 | 30.83 | 12.97 |
| 348 | 40.16 | 29.50 | 10.66 | 760 | 45.62 | 33.77 | 11.84 | 661 | 27.40 | 15.20 | 12.20 |
| 654 | 30.91 | 20.49 | 10.42 | 816 | 16.24 | 5.12 | 11.11 | 366 | 24.87 | 13.08 | 11.78 |
| 366 | 27.95 | 17.72 | 10.24 | 585 | 14.95 | 4.31 | 10.64 | 585 | 13.27 | 1.76 | 11.51 |
| 730 | 23.62 | 13.65 | 9.98 | 730 | 22.16 | 11.70 | 10.47 | 201 | 30.69 | 19.69 | 11.01 |
| 260 | 45.67 | 35.80 | 9.87 | 578 | 66.24 | 56.06 | 10.17 | 393 | 39.86 | 28.95 | 10.91 |
| 196 | 10.24 | 1.60 | 8.63 | 201 | 31.27 | 22.54 | 8.73 | 374 | 33.50 | 23.86 | 9.63 |
| 542 | 79.53 | 71.07 | 8.46 | 393 | 40.38 | 31.81 | 8.57 | 816 | 12.89 | 4.36 | 8.53 |
| 393 | 40.35 | 33.60 | 6.75 | 429 | 8.16 | 1.49 | 6.67 | 578 | 63.43 | 55.06 | 8.37 |
| 649 | 9.84 | 4.34 | 5.50 | 487 | 14.35 | 9.39 | 4.95 | 263 | 33.55 | 27.32 | 6.23 |
| 751 | 7.28 | 1.97 | 5.31 | 604 | 8.08 | 3.25 | 4.83 | 429 | 6.26 | 0.85 | 5.41 |
| 441 | 37.40 | 32.20 | 5.20 | 197 | 6.01 | 1.41 | 4.60 | 632 | 14.24 | 9.33 | 4.91 |
| 631 | 8.66 | 3.94 | 4.72 | 14 | 74.14 | 69.60 | 4.54 | 197 | 5.83 | 1.15 | 4.67 |
| 573 | 10.04 | 5.44 | 4.60 | 482 | 9.88 | 6.19 | 3.69 | 487 | 13.05 | 8.66 | 4.39 |
| 262 | 10.43 | 6.31 | 4.13 | 631 | 7.04 | 3.42 | 3.63 | 828 | 9.82 | 6.06 | 3.76 |
| 515 | 8.46 | 4.87 | 3.59 | 341 | 8.25 | 4.70 | 3.55 | 604 | 6.58 | 2.85 | 3.73 |
| 545 | 12.40 | 8.84 | 3.56 | 374 | 31.27 | 27.75 | 3.52 | 367 | 75.84 | 72.14 | 3.70 |
| 429 | 6.50 | 3.20 | 3.29 | 632 | 14.00 | 10.89 | 3.12 | 208 | 6.31 | 2.91 | 3.40 |
| 530 | 29.33 | 26.23 | 3.10 | 208 | 6.79 | 3.67 | 3.11 | 544 | 46.39 | 43.31 | 3.08 |
| 585 | 10.43 | 7.34 | 3.09 | 263 | 32.65 | 29.68 | 2.97 | 476 | 6.90 | 4.00 | 2.91 |
| 201 | 27.95 | 24.96 | 2.99 | 573 | 8.08 | 5.17 | 2.91 | 341 | 7.01 | 4.60 | 2.41 |
| 524 | 6.30 | 3.77 | 2.53 | 260 | 39.09 | 36.42 | 2.67 | 262 | 8.04 | 5.63 | 2.40 |
| 4 | 62.20 | 59.96 | 2.24 | 828 | 9.45 | 7.39 | 2.06 | 678 | 7.98 | 5.69 | 2.29 |
| 759 | 3.35 | 1.50 | 1.84 | 262 | 8.16 | 6.28 | 1.88 | 260 | 38.24 | 36.04 | 2.20 |
| 19 | 4.13 | 2.30 | 1.83 | 751 | 3.95 | 2.13 | 1.82 | 19 | 3.56 | 1.45 | 2.11 |
| 604 | 6.10 | 4.60 | 1.50 | 19 | 3.69 | 2.01 | 1.69 | 649 | 6.09 | 4.06 | 2.04 |
| 341 | 7.09 | 5.64 | 1.45 | 430 | 1.98 | 0.30 | 1.68 | 573 | 7.07 | 5.09 | 1.98 |
| 847 | 1.38 | 0.03 | 1.34 | 716 | 26.98 | 25.36 | 1.61 | 631 | 5.50 | 3.63 | 1.87 |
| 539 | 3.74 | 2.50 | 1.24 | 411 | 3.44 | 2.26 | 1.17 | 5 | 6.31 | 4.97 | 1.34 |
| 430 | 1.57 | 0.70 | 0.87 | 649 | 5.84 | 4.70 | 1.15 | 751 | 3.34 | 2.06 | 1.28 |
| 723 | 21.65 | 20.79 | 0.87 | 759 | 2.32 | 1.49 | 0.83 | 430 | 1.40 | 0.24 | 1.16 |
| 339 | 1.77 | 0.97 | 0.80 | 561 | 1.29 | 0.56 | 0.73 | 4 | 61.00 | 59.90 | 1.10 |
| 463 | 3.74 | 3.07 | 0.67 | 847 | 0.69 | 0.00 | 0.69 | 338 | 1.46 | 0.97 | 0.49 |
| 120 | 0.59 | 0.03 | 0.56 | 416 | 5.07 | 4.40 | 0.67 | 561 | 1.02 | 0.55 | 0.48 |
| 808 | 0.79 | 0.27 | 0.52 | 790 | 1.89 | 1.24 | 0.65 | 847 | 0.43 | 0.00 | 0.43 |
| 811 | 0.79 | 0.33 | 0.45 | 463 | 3.61 | 2.99 | 0.62 | 795 | 1.19 | 0.79 | 0.40 |
| 790 | 1.77 | 1.40 | 0.37 | 539 | 3.09 | 2.48 | 0.62 | 184 | 0.81 | 0.48 | 0.32 |
| 264 | 0.98 | 0.63 | 0.35 | 5 | 6.01 | 5.47 | 0.55 | 833 | 0.38 | 0.06 | 0.32 |
| 307 | 2.76 | 2.50 | 0.25 | 365 | 2.84 | 2.43 | 0.40 | 120 | 0.22 | 0.00 | 0.22 |
| 410 | 0.20 | 0.00 | 0.20 | 134 | 0.52 | 0.13 | 0.39 | 827 | 0.22 | 0.06 | 0.16 |
| 117 | 0.79 | 0.60 | 0.19 | 338 | 1.46 | 1.11 | 0.35 | 134 | 0.32 | 0.18 | 0.14 |
| 338 | 1.38 | 1.20 | 0.18 | 185 | 4.38 | 4.14 | 0.24 | 759 | 1.83 | 1.70 | 0.14 |
| 134 | 0.39 | 0.23 | 0.16 | 204 | 0.26 | 0.04 | 0.22 | 818 | 0.16 | 0.06 | 0.10 |
| 204 | 0.20 | 0.10 | 0.10 | 120 | 0.26 | 0.04 | 0.22 | 204 | 0.16 | 0.06 | 0.10 |
| 827 | 0.20 | 0.13 | 0.06 | 217 | 0.43 | 0.26 | 0.17 | 411 | 2.70 | 2.60 | 0.09 |
| 100 | 0.00 | 0.00 | 0.00 | 833 | 0.34 | 0.17 | 0.17 | 849 | 0.05 | 0.00 | 0.05 |
| 99 | 0.00 | 0.00 | 0.00 | 827 | 0.26 | 0.09 | 0.17 | 563 | 0.05 | 0.00 | 0.05 |
| 10 | 0.00 | 0.00 | 0.00 | 351 | 5.24 | 5.08 | 0.16 | 410 | 0.05 | 0.00 | 0.05 |
| 98 | 0.00 | 0.00 | 0.00 | 27 | 0.77 | 0.64 | 0.13 | 331 | 0.05 | 0.00 | 0.05 |
| 97 | 0.00 | 0.00 | 0.00 | 808 | 0.43 | 0.30 | 0.13 | 168 | 0.05 | 0.00 | 0.05 |
| 96 | 0.00 | 0.00 | 0.00 | 209 | 0.26 | 0.13 | 0.13 | 119 | 0.05 | 0.00 | 0.05 |
| 95 | 0.00 | 0.00 | 0.00 | 524 | 4.21 | 4.10 | 0.11 | 863 | 0.11 | 0.06 | 0.05 |
| 94 | 0.00 | 0.00 | 0.00 | 563 | 0.09 | 0.00 | 0.09 | 790 | 1.46 | 1.45 | 0.00 |
| 93 | 0.00 | 0.00 | 0.00 | 410 | 0.09 | 0.00 | 0.09 | 100 | 0.00 | 0.00 | 0.00 |
| 92 | 0.00 | 0.00 | 0.00 | 331 | 0.09 | 0.00 | 0.09 | 99 | 0.00 | 0.00 | 0.00 |
| 91 | 0.00 | 0.00 | 0.00 | 339 | 1.12 | 1.07 | 0.05 | 10 | 0.00 | 0.00 | 0.00 |
| 90 | 0.00 | 0.00 | 0.00 | 811 | 0.43 | 0.38 | 0.05 | 98 | 0.00 | 0.00 | 0.00 |
| 89 | 0.00 | 0.00 | 0.00 | 470 | 0.17 | 0.13 | 0.04 | 97 | 0.00 | 0.00 | 0.00 |
| 882 | 0.00 | 0.00 | 0.00 | 651 | 0.09 | 0.04 | 0.04 | 96 | 0.00 | 0.00 | 0.00 |
| 881 | 0.00 | 0.00 | 0.00 | 100 | 0.00 | 0.00 | 0.00 | 95 | 0.00 | 0.00 | 0.00 |
| 880 | 0.00 | 0.00 | 0.00 | 99 | 0.00 | 0.00 | 0.00 | 94 | 0.00 | 0.00 | 0.00 |
| 879 | 0.00 | 0.00 | 0.00 | 10 | 0.00 | 0.00 | 0.00 | 93 | 0.00 | 0.00 | 0.00 |
| 88 | 0.00 | 0.00 | 0.00 | 98 | 0.00 | 0.00 | 0.00 | 92 | 0.00 | 0.00 | 0.00 |
| 878 | 0.00 | 0.00 | 0.00 | 97 | 0.00 | 0.00 | 0.00 | 91 | 0.00 | 0.00 | 0.00 |
| 9 | 0.00 | 0.00 | 0.00 | 96 | 0.00 | 0.00 | 0.00 | 90 | 0.00 | 0.00 | 0.00 |
| 877 | 0.00 | 0.00 | 0.00 | 95 | 0.00 | 0.00 | 0.00 | 89 | 0.00 | 0.00 | 0.00 |
| 876 | 0.00 | 0.00 | 0.00 | 94 | 0.00 | 0.00 | 0.00 | 882 | 0.00 | 0.00 | 0.00 |
| 875 | 0.00 | 0.00 | 0.00 | 93 | 0.00 | 0.00 | 0.00 | 881 | 0.00 | 0.00 | 0.00 |
| 874 | 0.00 | 0.00 | 0.00 | 92 | 0.00 | 0.00 | 0.00 | 880 | 0.00 | 0.00 | 0.00 |
| 873 | 0.00 | 0.00 | 0.00 | 91 | 0.00 | 0.00 | 0.00 | 879 | 0.00 | 0.00 | 0.00 |
| 872 | 0.00 | 0.00 | 0.00 | 90 | 0.00 | 0.00 | 0.00 | 88 | 0.00 | 0.00 | 0.00 |
| 871 | 0.00 | 0.00 | 0.00 | 89 | 0.00 | 0.00 | 0.00 | 878 | 0.00 | 0.00 | 0.00 |
| 870 | 0.00 | 0.00 | 0.00 | 882 | 0.00 | 0.00 | 0.00 | 9 | 0.00 | 0.00 | 0.00 |
| 869 | 0.00 | 0.00 | 0.00 | 881 | 0.00 | 0.00 | 0.00 | 877 | 0.00 | 0.00 | 0.00 |
| 87 | 0.00 | 0.00 | 0.00 | 880 | 0.00 | 0.00 | 0.00 | 876 | 0.00 | 0.00 | 0.00 |
| 868 | 0.00 | 0.00 | 0.00 | 879 | 0.00 | 0.00 | 0.00 | 875 | 0.00 | 0.00 | 0.00 |
| 867 | 0.00 | 0.00 | 0.00 | 88 | 0.00 | 0.00 | 0.00 | 874 | 0.00 | 0.00 | 0.00 |
| 866 | 0.00 | 0.00 | 0.00 | 878 | 0.00 | 0.00 | 0.00 | 873 | 0.00 | 0.00 | 0.00 |
| 861 | 0.00 | 0.00 | 0.00 | 9 | 0.00 | 0.00 | 0.00 | 872 | 0.00 | 0.00 | 0.00 |
| 860 | 0.00 | 0.00 | 0.00 | 877 | 0.00 | 0.00 | 0.00 | 871 | 0.00 | 0.00 | 0.00 |
| 859 | 0.00 | 0.00 | 0.00 | 876 | 0.00 | 0.00 | 0.00 | 870 | 0.00 | 0.00 | 0.00 |
| 86 | 0.00 | 0.00 | 0.00 | 875 | 0.00 | 0.00 | 0.00 | 869 | 0.00 | 0.00 | 0.00 |
| 858 | 0.00 | 0.00 | 0.00 | 874 | 0.00 | 0.00 | 0.00 | 87 | 0.00 | 0.00 | 0.00 |
| 857 | 0.00 | 0.00 | 0.00 | 873 | 0.00 | 0.00 | 0.00 | 868 | 0.00 | 0.00 | 0.00 |
| 856 | 0.00 | 0.00 | 0.00 | 872 | 0.00 | 0.00 | 0.00 | 867 | 0.00 | 0.00 | 0.00 |
| 855 | 0.00 | 0.00 | 0.00 | 871 | 0.00 | 0.00 | 0.00 | 866 | 0.00 | 0.00 | 0.00 |
| 854 | 0.00 | 0.00 | 0.00 | 870 | 0.00 | 0.00 | 0.00 | 861 | 0.00 | 0.00 | 0.00 |
| 853 | 0.00 | 0.00 | 0.00 | 869 | 0.00 | 0.00 | 0.00 | 860 | 0.00 | 0.00 | 0.00 |
| 852 | 0.00 | 0.00 | 0.00 | 87 | 0.00 | 0.00 | 0.00 | 859 | 0.00 | 0.00 | 0.00 |
| 851 | 0.00 | 0.00 | 0.00 | 868 | 0.00 | 0.00 | 0.00 | 86 | 0.00 | 0.00 | 0.00 |
| 850 | 0.00 | 0.00 | 0.00 | 867 | 0.00 | 0.00 | 0.00 | 858 | 0.00 | 0.00 | 0.00 |
| 85 | 0.00 | 0.00 | 0.00 | 866 | 0.00 | 0.00 | 0.00 | 857 | 0.00 | 0.00 | 0.00 |

TableS3: **Best 100 negative Fingerprints in EGFR10, 100 and 1000 datasets**

| **EGFR10 Dataset** | | | | **EGFR100 Dataset** | | | | **EGFR1000 Dataset** | | | |
| --- | --- | --- | --- | --- | --- | --- | --- | --- | --- | --- | --- |
| ***FP No.*** | ***Freq. (+)*** | ***Freq. (-)*** | ***Difference*** | ***FP No.*** | ***Freq. (+)*** | ***Freq. (-)*** | ***Difference*** | ***FP No.*** | ***Freq. (+)*** | ***Freq. (-)*** | ***Difference*** |
| 698 | 21.26 | 45.15 | -23.89 | 698 | 23.20 | 50.85 | -27.66 | 686 | 11.97 | 38.58 | -26.61 |
| 673 | 8.27 | 31.80 | -23.53 | 694 | 8.59 | 30.32 | -21.72 | 698 | 30.20 | 54.63 | -24.43 |
| 690 | 57.48 | 76.51 | -19.03 | 714 | 3.78 | 25.02 | -21.24 | 714 | 7.17 | 29.98 | -22.81 |
| 700 | 19.29 | 38.00 | -18.71 | 690 | 63.83 | 78.78 | -14.95 | 145 | 34.79 | 52.39 | -17.60 |
| 714 | 3.54 | 20.42 | -16.88 | 669 | 3.95 | 18.74 | -14.79 | 711 | 69.26 | 85.58 | -16.33 |
| 145 | 30.31 | 45.28 | -14.96 | 145 | 33.42 | 48.04 | -14.62 | 619 | 10.95 | 26.04 | -15.10 |
| 701 | 14.37 | 28.50 | -14.13 | 700 | 25.95 | 39.97 | -14.02 | 295 | 13.75 | 27.62 | -13.87 |
| 669 | 2.17 | 15.92 | -13.75 | 422 | 38.06 | 50.00 | -11.94 | 700 | 29.13 | 42.28 | -13.15 |
| 195 | 6.50 | 18.02 | -11.52 | 152 | 8.42 | 18.92 | -10.50 | 152 | 9.39 | 22.23 | -12.84 |
| 382 | 2.56 | 11.61 | -9.05 | 361 | 6.10 | 16.48 | -10.38 | 687 | 4.91 | 16.96 | -12.05 |
| 21 | 45.87 | 54.82 | -8.96 | 687 | 4.12 | 13.88 | -9.75 | 442 | 3.29 | 13.14 | -9.85 |
| 152 | 7.87 | 16.72 | -8.84 | 302 | 11.17 | 20.45 | -9.28 | 302 | 12.84 | 22.35 | -9.51 |
| 697 | 2.36 | 11.04 | -8.68 | 821 | 7.47 | 16.40 | -8.92 | 697 | 5.34 | 14.78 | -9.44 |
| 302 | 10.04 | 18.62 | -8.58 | 382 | 4.47 | 13.11 | -8.64 | 453 | 32.31 | 41.55 | -9.24 |
| 361 | 5.71 | 14.25 | -8.54 | 340 | 8.25 | 16.48 | -8.23 | 821 | 9.06 | 18.29 | -9.23 |
| 295 | 12.99 | 21.49 | -8.50 | 376 | 49.40 | 57.51 | -8.12 | 22 | 11.38 | 20.53 | -9.15 |
| 22 | 8.46 | 16.95 | -8.49 | 477 | 5.33 | 12.98 | -7.65 | 675 | 2.43 | 11.45 | -9.02 |
| 340 | 6.69 | 14.95 | -8.26 | 778 | 2.75 | 9.74 | -6.99 | 778 | 3.29 | 12.05 | -8.76 |
| 821 | 6.69 | 14.65 | -7.96 | 701 | 21.82 | 28.78 | -6.96 | 674 | 22.65 | 30.47 | -7.81 |
| 376 | 48.23 | 55.76 | -7.53 | 675 | 2.15 | 8.97 | -6.82 | 361 | 9.39 | 17.08 | -7.70 |
| 687 | 4.53 | 11.75 | -7.22 | 195 | 11.43 | 18.23 | -6.81 | 820 | 2.00 | 9.57 | -7.57 |
| 820 | 0.59 | 6.47 | -5.88 | 820 | 1.12 | 7.86 | -6.74 | 340 | 10.19 | 17.75 | -7.55 |
| 475 | 3.35 | 9.21 | -5.86 | 697 | 5.58 | 11.91 | -6.33 | 701 | 22.92 | 30.28 | -7.36 |
| 421 | 3.94 | 9.24 | -5.31 | 22 | 11.86 | 17.63 | -5.78 | 376 | 51.40 | 58.63 | -7.23 |
| 675 | 2.17 | 7.44 | -5.28 | 402 | 6.19 | 11.91 | -5.73 | 475 | 4.96 | 12.17 | -7.21 |
| 577 | 1.18 | 6.44 | -5.26 | 703 | 1.80 | 7.34 | -5.54 | 21 | 50.22 | 57.36 | -7.14 |
| 627 | 0.20 | 5.14 | -4.94 | 151 | 1.80 | 7.22 | -5.41 | 195 | 13.32 | 19.69 | -6.36 |
| 683 | 0.79 | 5.61 | -4.82 | 683 | 1.55 | 6.49 | -4.94 | 402 | 7.07 | 13.33 | -6.26 |
| 40 | 0.98 | 5.64 | -4.65 | 399 | 5.41 | 10.33 | -4.92 | 151 | 1.29 | 7.33 | -6.03 |
| 151 | 0.20 | 4.80 | -4.61 | 40 | 1.72 | 6.49 | -4.77 | 703 | 2.86 | 8.42 | -5.56 |
| 778 | 3.74 | 8.04 | -4.30 | 627 | 1.29 | 5.89 | -4.60 | 36 | 1.35 | 6.90 | -5.56 |
| 779 | 22.44 | 26.63 | -4.19 | 835 | 0.86 | 5.34 | -4.48 | 627 | 1.94 | 7.03 | -5.08 |
| 186 | 0.39 | 4.54 | -4.14 | 533 | 10.57 | 14.94 | -4.38 | 530 | 24.33 | 29.07 | -4.75 |
| 370 | 2.56 | 6.61 | -4.05 | 648 | 8.93 | 13.07 | -4.13 | 399 | 6.47 | 11.21 | -4.73 |
| 835 | 0.39 | 4.44 | -4.04 | 21 | 50.95 | 54.95 | -4.01 | 425 | 1.08 | 5.63 | -4.55 |
| 425 | 0.00 | 3.77 | -3.77 | 471 | 0.00 | 3.80 | -3.80 | 683 | 2.70 | 7.21 | -4.51 |
| 684 | 25.79 | 29.53 | -3.74 | 425 | 0.69 | 4.48 | -3.80 | 835 | 1.78 | 6.12 | -4.34 |
| 510 | 3.74 | 7.21 | -3.47 | 707 | 0.86 | 4.61 | -3.75 | 202 | 1.56 | 5.69 | -4.13 |
| 399 | 5.91 | 9.18 | -3.27 | 220 | 0.00 | 3.63 | -3.63 | 520 | 0.65 | 4.72 | -4.08 |
| 761 | 0.00 | 3.27 | -3.27 | 520 | 0.17 | 3.76 | -3.59 | 707 | 1.51 | 5.45 | -3.94 |
| 434 | 3.35 | 6.54 | -3.19 | 421 | 6.10 | 9.65 | -3.55 | 719 | 0.54 | 4.06 | -3.52 |
| 15 | 1.97 | 5.14 | -3.17 | 761 | 0.52 | 3.84 | -3.33 | 689 | 0.22 | 3.69 | -3.48 |
| 416 | 1.97 | 5.04 | -3.07 | 337 | 2.92 | 6.11 | -3.18 | 637 | 1.40 | 4.85 | -3.44 |
| 695 | 0.98 | 4.04 | -3.05 | 530 | 24.57 | 27.71 | -3.14 | 648 | 10.14 | 13.57 | -3.43 |
| 520 | 0.00 | 3.00 | -3.00 | 347 | 60.82 | 63.66 | -2.84 | 799 | 7.93 | 11.27 | -3.34 |
| 471 | 0.00 | 2.97 | -2.97 | 719 | 0.34 | 3.12 | -2.77 | 40 | 3.51 | 6.66 | -3.16 |
| 648 | 9.25 | 12.21 | -2.96 | 637 | 1.20 | 3.97 | -2.77 | 510 | 5.29 | 8.30 | -3.01 |
| 215 | 0.39 | 3.34 | -2.94 | 652 | 0.00 | 2.56 | -2.56 | 370 | 4.64 | 7.63 | -2.99 |
| 337 | 2.56 | 5.44 | -2.88 | 611 | 1.12 | 3.54 | -2.43 | 215 | 1.67 | 4.30 | -2.63 |
| 781 | 10.04 | 12.91 | -2.87 | 202 | 1.72 | 3.89 | -2.17 | 781 | 11.33 | 13.81 | -2.48 |
| 799 | 7.09 | 9.88 | -2.79 | 510 | 5.33 | 7.43 | -2.10 | 186 | 2.80 | 5.27 | -2.46 |
| 614 | 2.76 | 5.34 | -2.58 | 781 | 11.17 | 13.19 | -2.03 | 337 | 3.88 | 6.30 | -2.42 |
| 719 | 0.00 | 2.57 | -2.57 | 769 | 0.69 | 2.56 | -1.87 | 400 | 4.58 | 6.90 | -2.32 |
| 402 | 7.87 | 10.38 | -2.50 | 731 | 0.60 | 2.39 | -1.79 | 611 | 1.62 | 3.94 | -2.32 |
| 611 | 0.59 | 3.07 | -2.48 | 146 | 0.17 | 1.92 | -1.75 | 434 | 5.02 | 7.33 | -2.31 |
| 664 | 12.20 | 14.51 | -2.31 | 184 | 0.77 | 2.52 | -1.75 | 480 | 0.05 | 2.36 | -2.31 |
| 731 | 0.00 | 2.10 | -2.10 | 480 | 0.00 | 1.71 | -1.71 | 307 | 1.51 | 3.69 | -2.18 |
| 652 | 0.00 | 2.00 | -2.00 | 741 | 0.43 | 2.13 | -1.71 | 157 | 0.05 | 2.12 | -2.07 |
| 637 | 1.38 | 3.34 | -1.96 | 717 | 0.17 | 1.84 | -1.66 | 23 | 0.49 | 2.36 | -1.88 |
| 146 | 0.00 | 1.74 | -1.74 | 157 | 0.00 | 1.54 | -1.54 | 688 | 0.76 | 2.54 | -1.79 |
| 411 | 1.18 | 2.87 | -1.69 | 23 | 0.34 | 1.88 | -1.54 | 741 | 0.70 | 2.48 | -1.78 |
| 26 | 2.76 | 4.40 | -1.65 | 720 | 0.09 | 1.58 | -1.49 | 499 | 0.86 | 2.60 | -1.74 |
| 23 | 0.00 | 1.60 | -1.60 | 450 | 0.17 | 1.67 | -1.49 | 720 | 0.27 | 2.00 | -1.73 |
| 769 | 0.59 | 2.17 | -1.58 | 159 | 1.03 | 2.52 | -1.49 | 713 | 0.16 | 1.88 | -1.72 |
| 741 | 0.39 | 1.77 | -1.37 | 688 | 0.69 | 2.05 | -1.36 | 769 | 1.13 | 2.85 | -1.71 |
| 480 | 0.00 | 1.33 | -1.33 | 713 | 0.09 | 1.41 | -1.32 | 450 | 0.38 | 2.06 | -1.68 |
| 161 | 0.20 | 1.47 | -1.27 | 740 | 1.55 | 2.82 | -1.27 | 717 | 0.54 | 2.12 | -1.58 |
| 717 | 0.20 | 1.47 | -1.27 | 614 | 4.21 | 5.34 | -1.13 | 665 | 3.24 | 4.78 | -1.55 |
| 633 | 1.18 | 2.44 | -1.25 | 490 | 3.69 | 4.74 | -1.05 | 614 | 4.26 | 5.75 | -1.49 |
| 157 | 0.00 | 1.20 | -1.20 | 633 | 1.55 | 2.56 | -1.02 | 761 | 2.16 | 3.57 | -1.42 |
| 450 | 0.20 | 1.33 | -1.14 | 722 | 0.00 | 0.98 | -0.98 | 467 | 3.24 | 4.60 | -1.37 |
| 713 | 0.00 | 1.13 | -1.13 | 406 | 0.43 | 1.41 | -0.98 | 740 | 1.73 | 3.09 | -1.36 |
| 496 | 0.59 | 1.67 | -1.08 | 400 | 5.07 | 6.02 | -0.95 | 722 | 0.05 | 1.33 | -1.28 |
| 406 | 0.20 | 1.23 | -1.04 | 650 | 0.00 | 0.90 | -0.90 | 161 | 0.70 | 1.94 | -1.24 |
| 720 | 0.20 | 1.23 | -1.04 | 499 | 1.12 | 2.01 | -0.89 | 650 | 0.05 | 1.21 | -1.16 |
| 347 | 61.81 | 62.83 | -1.02 | 528 | 0.00 | 0.85 | -0.85 | 528 | 0.05 | 1.15 | -1.10 |
| 732 | 0.20 | 1.13 | -0.94 | 767 | 0.26 | 1.11 | -0.85 | 590 | 0.00 | 0.85 | -0.85 |
| 624 | 0.20 | 1.00 | -0.80 | 485 | 1.46 | 2.31 | -0.85 | 660 | 7.71 | 8.54 | -0.83 |
| 722 | 0.00 | 0.77 | -0.77 | 799 | 8.93 | 9.78 | -0.84 | 696 | 0.16 | 0.97 | -0.81 |
| 767 | 0.20 | 0.93 | -0.74 | 724 | 0.09 | 0.90 | -0.81 | 494 | 0.54 | 1.33 | -0.79 |
| 561 | 0.20 | 0.90 | -0.70 | 369 | 0.17 | 0.94 | -0.77 | 146 | 1.13 | 1.88 | -0.74 |
| 650 | 0.00 | 0.70 | -0.70 | 862 | 0.00 | 0.73 | -0.73 | 671 | 0.00 | 0.73 | -0.73 |
| 740 | 1.77 | 2.47 | -0.70 | 725 | 0.09 | 0.81 | -0.73 | 862 | 0.16 | 0.85 | -0.69 |
| 528 | 0.00 | 0.67 | -0.67 | 494 | 0.43 | 1.15 | -0.72 | 731 | 1.46 | 2.12 | -0.66 |
| 725 | 0.00 | 0.67 | -0.67 | 590 | 0.00 | 0.60 | -0.60 | 565 | 0.38 | 1.03 | -0.65 |
| 5 | 5.12 | 5.77 | -0.65 | 434 | 5.76 | 6.28 | -0.52 | 725 | 0.27 | 0.91 | -0.64 |
| 494 | 0.39 | 1.00 | -0.61 | 671 | 0.00 | 0.51 | -0.51 | 118 | 0.22 | 0.85 | -0.63 |
| 263 | 30.12 | 30.70 | -0.58 | 732 | 0.69 | 1.20 | -0.51 | 767 | 0.54 | 1.15 | -0.61 |
| 27 | 0.20 | 0.77 | -0.57 | 298 | 0.00 | 0.47 | -0.47 | 777 | 0.00 | 0.61 | -0.61 |
| 369 | 0.20 | 0.77 | -0.57 | 774 | 0.00 | 0.47 | -0.47 | 188 | 61.17 | 61.72 | -0.56 |
| 862 | 0.00 | 0.57 | -0.57 | 777 | 0.00 | 0.43 | -0.43 | 298 | 0.05 | 0.61 | -0.55 |
| 724 | 0.20 | 0.70 | -0.50 | 300 | 0.17 | 0.60 | -0.43 | 490 | 4.15 | 4.66 | -0.51 |
| 590 | 0.00 | 0.47 | -0.47 | 696 | 0.26 | 0.68 | -0.43 | 738 | 0.16 | 0.67 | -0.50 |
| 208 | 4.33 | 4.77 | -0.44 | 415 | 0.00 | 0.38 | -0.38 | 415 | 0.05 | 0.48 | -0.43 |
| 671 | 0.00 | 0.40 | -0.40 | 132 | 0.00 | 0.34 | -0.34 | 27 | 0.49 | 0.91 | -0.42 |
| 118 | 0.20 | 0.57 | -0.37 | 738 | 0.17 | 0.51 | -0.34 | 724 | 0.43 | 0.85 | -0.42 |
| 298 | 0.00 | 0.37 | -0.37 | 186 | 5.33 | 5.64 | -0.31 | 365 | 2.37 | 2.79 | -0.41 |
| 774 | 0.00 | 0.37 | -0.37 | 512 | 0.09 | 0.38 | -0.30 | 300 | 0.27 | 0.67 | -0.40 |
| 777 | 0.00 | 0.33 | -0.33 | 624 | 0.69 | 0.98 | -0.29 | 132 | 0.05 | 0.42 | -0.37 |
| 184 | 0.39 | 0.70 | -0.31 | 118 | 0.34 | 0.60 | -0.25 | 762 | 0.05 | 0.42 | -0.37 |

*****abbreviations: FP no. PubChem fingerprint Number, Freq = Frequency, + = active compounds, - = inactive compounds, Acc. = Accuracy, MCC = Matthew’s correlation coefficient, ROC = Receiver operating characteristic.
